# Genome–Environment Interactions and Psychiatric Disorders

**DOI:** 10.3390/biomedicines11041209

**Published:** 2023-04-19

**Authors:** Jacob Peedicayil

**Affiliations:** Department of Pharmacology & Clinical Pharmacology, Christian Medical College, Vellore 632 002, India; jpeedi@cmcvellore.ac.in; Tel.: +91-0416-2284237

**Keywords:** genetic, genome, environment, epigenetic, psychiatric disorder

## Abstract

Environmental factors are known to interact with the genome by altering epigenetic mechanisms regulating gene expression and contributing to the pathogenesis of psychiatric disorders. This article is a narrative review of how the major environmental factors contribute to the pathogenesis of common psychiatric disorders such as schizophrenia, bipolar disorder, major depressive disorder, and anxiety disorder this way. The cited articles were published between 1 January 2000 and 31 December 2022 and were obtained from PubMed and Google Scholar. The search terms used were as follows: gene or genetic; genome; environment; mental or psychiatric disorder; epigenetic; and interaction. The following environmental factors were found to act epigenetically on the genome to influence the pathogenesis of psychiatric disorders: social determinants of mental health, maternal prenatal psychological stress, poverty, migration, urban dwelling, pregnancy and birth complications, alcohol and substance abuse, microbiota, and prenatal and postnatal infections. The article also discusses the ways by which factors such as drugs, psychotherapy, electroconvulsive therapy, and physical exercise act epigenetically to alleviate the symptoms of psychiatric disorders in affected patients. These data will be useful information for clinical psychiatrists and those researching the pathogenesis and treatment of psychiatric disorders.

## 1. Introduction

Psychiatric disorders include the following [1]: schizophrenia (SZ) and other primary psychotic disorders; mood disorders such as bipolar disorder (BD) and major depressive disorder (MDD); anxiety and fear-related disorders; obsessive -compulsive and related disorders; and personality disorders. It has been well established from family, twin, and adoption studies that the psychosis SZ, the mood disorders BD and MDD, and anxiety disorder (AD) have a genetic basis. These are common, chronic disorders whose inheritance patterns involve several genes, possibly hundreds, or even thousands.

Environmental factors are well known to interact with the genome and influence the pathogenesis of psychiatric disorders [2]. This purpose of this article is to present a narrative review of the genome–environment interactions underlying common psychiatric disorders such as SZ, BD, MDD, and AD whose pathogeneses continue to be unclear, and which continue to cause much suffering to affected patients, despite a large amount of research into finding better treatments. The guidelines of Murphy [3] were followed while writing the review. It was thought that a proper understanding of how the genome and the environment interact in these disorders may help in the development of biomarkers for these disorders and improve the treatment of these disorders. The cited articles were obtained from PubMed and Google Scholar and the period of review chosen was from 1 January 2000 to 31 December 2022. The search terms used were as follows: gene or genetic; genome; environment; mental or psychiatric disorder; epigenetic; and interaction. Both preclinical and clinical studies were included. Both original studies and review articles were included. Case reports and letters to the editor were excluded. If there were multiple papers dealing with the relevant topic, the paper or papers that best described the genome–environment interactions involved were chosen. Non-English, and non-peer-reviewed papers were excluded.

## 2. Genetic Basis of Psychiatric Disorders

It has been estimated that about 70 to 80% of the approximately 25,000 genes of the human genome are expressed in the brain, and because most genes encode more than one protein, there may be 100,000 different proteins in the brain [4]. Of these, about 10,000 are known proteins and 100 or less of these are targets for currently used psychotropic drugs [4]. The study of families using population genetics methods over the last 50 years has consistently supported a genetic and heritable component to psychiatric disorders. More recently, molecular genetic techniques have shown that specific regions on chromosomes are associated with specific diagnoses. Many genes, including those encoding proteins involved in synaptic transmission, have been found to correlate with psychiatric disorders [4]. However, much more work is needed to determine precisely how these genes predispose individuals to psychiatric disease states.

## 3. Epigenetic Mechanisms of Gene Expression

Epigenetics, above or in addition to genetics, is a very active area of biomedical research. There are five major epigenetic mechanisms of regulation of gene expression [5]. These are DNA methylation, histone modifications, non-coding RNA (ncRNA)-mediated regulation of gene expression, histone variants, and chromatin remodeling [6]. Of the epigenetic mechanisms, the most and best studied has been DNA methylation. DNA becomes methylated mainly at cytosine bases located on CpG dinucleotides giving rise to 5-methylcytosine (5mC) [6,7]. This reaction is catalyzed by the activity of the de novo DNA methyltransferases Dnmt3a, Dnmt3b, and Dnmt3L. Preservation (or maintenance) of this methylation is carried out by the maintenance methyltransferase Dnmt1 [8]. DNA methylation is of major importance for mammalian development. It is also involved in the repression of transposons and genes, but is also associated with actively transcribed gene bodies, and in some situations, with gene activation [8]. Another epigenetic mechanism biochemically related to DNA methylation is mediated by 5-hydroxymethylcytosine (5-hmC) which is formed by the oxidation of the methyl group of 5mC, a reaction catalyzed by the Ten-Eleven-Translocation (TET) enzymes [9]. Although most work on TET enzymes has been performed in embryonic stem cells, the highest levels of 5-hmC are found in brain neurons, suggesting a role for this epigenetic mediator in the regulation of neuronal differentiation, neural plasticity, and brain function [9]. Chromatin comprises repeating units of nucleosomes, which contain two copies of each of the four different histones with approximately 200 base pairs of DNA [10]. The four histones are called H2A, H2B, H3, and H4. Additionally, there is a linker histone called H1. The histones can be changed post-translationally by biochemical reactions such as acetylation, phosphorylation, and methylation, which result in changes in gene transcription [10]. A further mechanism for epigenetic regulation of gene expression is non-coding RNA (ncRNA)-mediated regulation of gene expression. ncRNAs have emerged as indispensable players in the diagnosis, development, and treatment of virtually every abnormality concerning physiology, pathology, genetics, epigenetics, oncology, and developmental disorders [11]. ncRNAs include microRNAs (miRNAs), small nuclear RNAs (snRNAs), small nucleolar RNAs (snoRNAs), long non-coding RNAs (lncRNAs), and circular RNAs (circRNAs) [12]. In addition to the core histones mentioned above, there are other histones which differ in amino acid sequence from the core histones called histone variants. Several histone variants exist for H2A, H2B, and H3. However, only one exists for H3 [13]. Histone variants may provide specific binding sites for nucleosome binding factors at certain genomic loci [13]. The fifth epigenetic mechanism, chromatin remodeling, can occur due to induction of chromatin-remodeling complexes which use energy from hydrolysis of ATP to change chromatin and nucleosome composition in a non-covalent manner [5]. The DNA sequence of genes and epigenetic mechanisms of gene expression are inextricably connected [14]. The sequence of bases in DNA can influence DNA methylation patterns [15]. Chromatin states can affect transcription factor binding, and DNA sequence polymorphism influences chromatin state [14]. Chromatin and DNA methylation demonstrate much variation in humans, and they control genome stability and mutability [14]. 

## 4. Epigenetic Mechanisms and Environmental Factors

The environment cannot interact with the genome by altering the DNA sequence easily. However, the environment can relatively easily change expression of the genome by affecting epigenetic mechanisms regulating gene expression [16,17,18]. Different epigenetic mechanisms can transmit the effects of the environment on the genome. For instance, in rats it was shown that the chemical vinclozilin methoxychlor reduces sperm count and motility by changing methylation of DNA in the testes [18]. It has been demonstrated that air pollution can change histones to cause changes in blood leukocytes [18]. The roundworm *Caenorhabditis elegans* has to differentiate deleterious from useful bacterial foods among the many bacteria it is exposed to in the surroundings. Kaletsky et al. [19] demonstrated that one exposure to purified small RNAs obtained from *Pseudomonas Aeuginosa* (PA14) is adequate to cause pathogen avoidance, both in the treated organisms as well as in four later generations of offspring. One *Pseudomonas aeruginosa* ncRNA, P11, is both needed and sufficient to convey learned avoidance of PA14, and its *Caenorhabditis elegans* target, maco-1, is necessary for avoidance.

## 5. Environmental Factors and Psychiatric Disorders

The common, chronic psychiatric disorders such as SZ, BD, and MDD have been described as suffering from the “curse of polygenicity” [20]. Indeed, there is evidence that such disorders may be “omnigenic”, that is, affecting all genes in the genome [21,22]. Why do common, chronic disorders such as these psychiatric disorders tend to be polygenic, or even omnigenic? The answer could be that in order to account for extensive environmental involvement, hundreds, or even thousands, of genes need to be involved [23].

## 6. Environmental Factors Acting Epigenetically in Psychiatric Disorders

Given below is evidence for several environmental factors altering epigenetic mechanisms of gene expression and leading to the development of psychiatric disorders (Table 1).

### 6.1. Social Determinants of Mental Health Including Early Life Stress and Adversity

Social determinants of mental health are characteristics or facets that affect individuals in relation to their social environment and how these affect mental health [24]. Examples of social determinants are social support, loneliness, marriage status, social disruption, bereavement, work environment, and social status [25]. Since the times of the work of the pioneers of psychiatry and psychology such as Sigmund Freud (1856–1939), Carl Jung (1875–1961), and Erik Erikson (1902–1994), it has been known that social determinants of mental health are involved in the pathogenesis of psychiatric disorders. In the 1950s, the British psychiatrist George Brown developed the concept of expressed emotion, which refers to the quality of family interactions, especially the existence of hostility, criticism, and emotional over-involvement with regard to a patient with a psychiatric disorder [26]. It has been shown that expressed emotion is a significant and robust predictor of relapse in a broad range of psychopathological conditions [27]. Michael Meaney, Moshe Szyf, and co-workers [28] demonstrated in 2004 using rats that increased licking and grooming of pups (LG) and arched back nursing (ABN) by mother rats changes the epigenome of the offspring at a glucocorticoid receptor (GR) gene promoter in the hippocampus of the rat pups. The pups of mothers that showed high LG and ABN had differing patterns of DNA methylation from those of mothers that showed low LG and ABN. The differences were first seen during the first week of infancy, became abrogated with cross-fostering, persisted into adult life, and correlated with changed histone acetylation and transcription factor (NGF-1A) binding to the promoter of the GR. The central infusion of the histone deacetylase inhibitor trichostatin A reduced the histone acetylation, DNA methylation, NGF-1A binding, altered GR expression, and the maternal effect of stress in the pups. In 2009, the same group including Gustavo Turecki extended these data from rats to humans [29]. The authors investigated DNA methylation differences at the same promoter between post-mortem hippocampus samples obtained from suicide victims with a history of childhood abuse and those from suicide victims without childhood abuse or controls. The authors found reduced levels of GR mRNA and mRNA transcripts having the GR 1F splice variant, and raised DNA methylation of the same promoter as in the study on rats. Based on such data, it was theorized that social determinants of mental health act via epigenetic mechanisms and contribute to the pathogenesis of psychiatric disorders [30,31], and it has been suggested that epigenetics is a link between social determinants of mental health and psychiatric disorders [32,33,34]. After the pioneering work by Meaney and Szyf, there have been many other studies conducted on animal models of psychiatric disorders, post-mortem human brain tissues, and peripheral tissues of psychiatric patients that have shown that social determinants of mental health act epigenetically to contribute to psychiatric disorders [35,36,37]. Interestingly, there is growing evidence that the mechanism of action of psychotherapy in the treatment of psychiatric disorders is by altering epigenetic changes [38]. In this context, it has been suggested that psychotherapy “undoes” what the disease has done to patients via epigenetic mechanisms [35].

#### Mechanism of Action of Social Determinants of Mental Health

**Figure 1:**
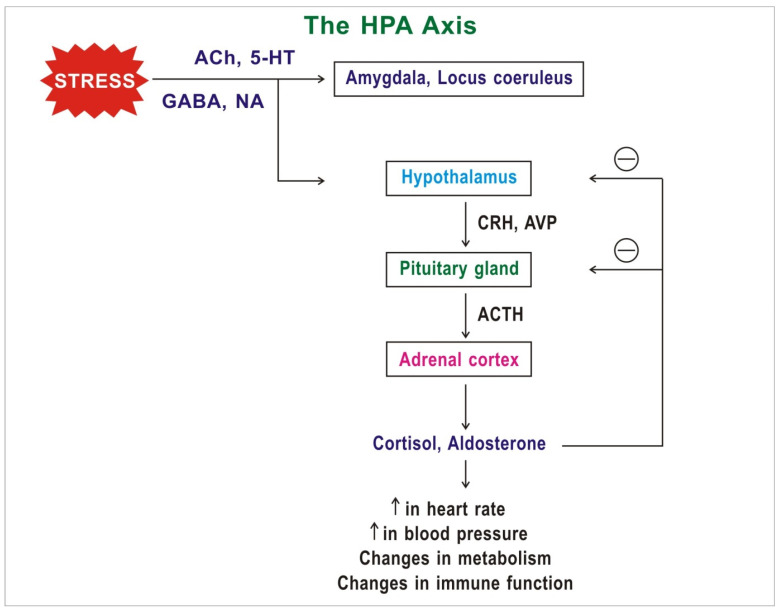
Diagram and simplified representation of the components of the hypothalamic–pituitary–adrenal (HPA) axis. Abbreviations: ACh: acetylcholine; ACTH: adrenocorticotropic hormone; AVP: arginine vasopressin; CRH: corticotropin-releasing hormone; GABA: gamma-aminobutyric acid; NA: noradrenaline.

The central pathway that takes part in the response to stress is the hypothalamic–pituitary–adrenal (HPA) axis (Figure 1). In 1914 the noted American physiologist Walter Cannon proposed the “fight or flight” model, which describes the body’s response to stress [39]. In the 1950s the Hungarian-Canadian endocrinologist Hans Selye proposed general adaptation syndrome, according to which chronic stress can induce a nonspecific response in the body, such as increased heart rate and blood pressure [40]. During stress, the amygdala, hypothalamus, and parts of the brain stem such as the locus coeruleus, which make up the central parts of the stress response, are activated [39]. 

Neurotransmitters such as serotonin, glutamate, and GABA participate in this signal transmission. When the neuronal signal moves from the amygdala and locus coeruleus to the hypothalamus, the hypothalamus releases many neuropeptides such as arginine vasopressin (AVP) and corticotropin-releasing hormone (CRH). In response, the anterior lobe of the pituitary gland releases adrenocorticotropic hormone (ACTH). AVP acts in concert with CRH to contribute to the ACTH response. ACTH activates its receptors in the adrenal cortex to release the stress-related hormones glucocorticoids (mainly cortisol), and the mineralocorticoid aldosterone. These hormones take part in the response to stress comprising an increase in heart rate, blood pressure, and changes in metabolism and immune function. In addition to these agents, other neuropeptides and neurotrophic factors such as neuropeptide Y, dynorphin, oxytocin, and brain-derived neurotrophic factor (BDNF) also affect the HPA axis and take part in the response to stress [41].

FK-506 binding protein 5 (*FKBP5*) codes for the protein Fkbp51, a heat shock protein 90 kDa (Hsp90) co-chaperone, which is associated with several mood disorders [42]. Fkbp51, along with Hsp90, control function of the GR via a negative feedback short loop. This signaling pathway quickly restores homeostasis in the HPA axis after stress. The expression of *FKBP5* increases with age due to reduced DNA methylation. High levels of Fkbp51 are linked with GR resistance and reduced coping behavior [42]. 

How is the HPA axis activated by social determinants of mental health such as stress? As given above in Section 6.1, social determinants of mental health can activate the HPA axis by changing DNA methylation at the GR promoter in the rat offspring hypothalamus [28]. In this context, in the mouse brain it has been shown that early life stress (ELS) may influence expression of histone deacetylase enzymes (HDACs) that can lead to a more condensed chromatin structure [43]. An activity-dependent alteration in Mecp2 is thought to be involved in the long-term de-repression of *AVP* gene activity in response to maternal separation in mice. It has been shown that maternal separation results in the phosphorylation of Mecp2, and hence to dissociation from the promoter region of the murine *AVP* gene in the paraventricular nucleus [43,44]. Later, the Mecp2 binding site is demethylated, leading to a sustained transcriptional activation of AVP by decreased binding of the Mecp2 repressor complex [45]. It is felt that the early priming to demethylation by exposure to ELS is mediated by polycomb repressive complexes and the TET proteins that attract DNA methyltransferases and HDACs in order to make sure that the right methylation status of the locus occurs [45]. In addition, MeCP2 acts on genes involved in the regulation of the HPA axis. In this context, it has been shown that MeCP2 knockout mice have raised mRNA levels of FKBP5 gene, serum glucocorticoid-regulated kinase 1, as well other GR-responsive genes in the absence of markedly raised plasma glucocorticoid levels, indicating that MeCP2 can act as a regulator of the action of glucocorticoids in neurons [46]. MeCP2 also interacts with several other enzymes that modify chromatin depending on protein phosphorylation status, leading to specific control of transcription at a locus [47].

Social determinants of mental health can also directly influence Dnmt1, the maintenance methyltransferase. In adult mice, stress has been shown to regulate *DNMT* expression in a brain-region-specific way [48,49]. ELS has also been shown to influence the expression of HDACs [50]. In addition, stress can cause long-term changes due to the activation of specific transcription factors, which cause local changes in epigenetic patterns. For instance, binding of the transcription factor Sp-1 causes inhibition of de novo DNA methylation [51]. Another pathway that is believed to cause long-term epigenetic changes due to exposure to the environment is the expression of miRNAs and their later targeting of stress-related pathways such as the HPA axis [43]. ncRNAs such as miRNAs have been shown to take part in the response to stress. miRNA expression has been studied in post-mortem human brains obtained from depressed patients, in the brains of animals demonstrating depression-like behavior, and in peripheral tissues including blood, urine, and saliva. It has been demonstrated that miRNAs form highly correlated networks in the brains of patients with MDD that differ from healthy controls, indicating that miRNA networks can cause specific behavioral phenotypes. In rats it has been demonstrated that rats which show learned helplessness, a clinical phenotype indicative of suicide risk, have a diminished miRNA response in the frontal cortex to acute stress compared to controls, indicating that anomalous expression of miRNAs results in defects in the stress coping response [52].

### 6.2. Maternal Prenatal Psychological Stress 

Maternal prenatal stress such as anxiety, depression, and perceived stress is well known to predispose the offspring to psychiatric disorders such as AD, MDD, and SZ [53,54,55]. Many studies, conducted in rodents and humans, suggest that epigenetic mechanisms are involved. For instance, Matrisciano et al. [56] demonstrated that prenatal restraint stress in mice causes abnormalities in the DNA methylation network and in behaviors suggestive of a SZ-like phenotype. Nazzari et al. [57] studied the DNA methylation status of *NR3C1* and *SLC6A4* in Italian mothers and infants who experienced the lockdown during the COVID-19 pandemic during the three pregnancy trimesters. It was shown that mothers and infants who experienced the lockdown during the first trimester of pregnancy have reduced methylation of *NR3C1* and *SLC6A4* in comparison to their counterparts who experienced the lockdown during the second or third pregnancy trimesters.

### 6.3. Pregnancy and Birth Complications

Pregnancy and birth complications have been linked with an increased risk for SZ and mood disorders [58]. One explanation for this increased risk is dysregulated epigenetic mechanisms in the child. In this context, Palma-Gudiel et al. [59] investigated the cerebro-placental ratio (CPR), a hemodynamic parameter reflecting fetal adaptation to hypoxic conditions, in a sample of monozygotic monochorionic twins (60 subjects), some of them having a history of prenatal complications. The authors focused on the following data: epigenetic age acceleration and DNA methylation at genes chosen in the polygenic risk score (PRS) for SZ that were very much expressed in the placenta. The researchers found that reduced CPR determined during the third trimester of pregnancy correlated with epigenetic age deceleration. The investigation of DNA methylation at placentally expressed genes of the PRS for SZ demonstrated methylation at cg06793497 (in the *EP300* gene) to correlate with CPR. This gene codes for the protein p300 that controls the function of many genes in tissues all over the body. p300 has an essential role in regulating cell growth and division and stimulating cells to mature and differentiate. Hence, this study demonstrates that an improper environment during the third trimester of pregnancy correlates with developmental immaturity with regard to epigenetic age and reduced CpG methylation in a gene taking part in hypoxia response and SZ genetic liability.

### 6.4. Poverty

Poverty is well known to be a predisposing factor for several psychiatric disorders. Such disorders include the psychoses, mood disorders, suicide, and alcohol and substance abuse [60,61]. The mechanisms by which poverty predisposes individuals to psychiatric disorders include social insecurity, unemployment, social discrimination, illiteracy and poor education, malnutrition, lack of access to clean water, polluted living environments, inadequate housing, and a relatively high incidence of accidents [60,61]. There is evidence that poverty could at least partially act epigenetically to predispose humans to psychiatric disorders. For instance, Hoare and colleagues [62] showed that epigenetic age acceleration in young adolescents from households with a low income is associated with changes in brain morphology, poorer visual memory, and visual spatial acuity.

### 6.5. Migration

Migration from one country to another is known to increase the risk for several psychiatric disorders [63,64]. This increased risk has been consistently observed in several economically advanced countries including the United Kingdom, Germany, Netherlands, Denmark, France, and Italy [64]. The increased risk could be higher for migrant refugees than non-refugees, and may last beyond the first generation into the second and third generations [64]. There is accumulating evidence that dysregulated epigenetic mechanisms are involved in psychiatric disorders associated with migration. 

For instance, in Latin American immigrant mothers and children, the associations between social determinants of mental health and resilience factors, and targeted DNA methylation in the stress-related genes *FKBP5* and *SLCA4* were investigated in a study by Clausing and Non [65]. The study found an epigenetic pathway through which early adversity and ongoing stressful life events correlate with important regulatory regions of these two genes.

### 6.6. Urban Dwelling

Urban dwelling such as in cities and big towns is thought to be a predisposing factor for non-communicable lifestyle diseases. The reasons include increased food availability, decreased non-recreational physical activity, and increased psychosocial stress [66]. The non-communicable diseases include psychiatric disorders such as SZ [67], MDD [68], and suicidal behavior [68]. There is evidence that one of the factors involved in the increased risk is epigenetics. For instance, Beydoun et al. [69] comprehensively investigated the relationship between DNA methylation epigenetic age acceleration and depressive symptoms in a prospective study involving a biracial group of urban adults. The authors found that in the total population and among whites, there was a cross-relationship between two measures of epigenetic age acceleration using the Horvath algorithm and the domain of positive affect, suggesting that accelerated aging can influence the specific domain of depressive symptoms in an adverse way.

### 6.7. Alcohol Use

Alcohol is a very commonly consumed drug, with its use increasing worldwide. Alcohol use disorder (AUD) is a major psychiatric disorder with significant economic and social consequences. Both acute and chronic exposure to alcohol affect epigenetic mechanisms regulating gene expression in the brain and in the periphery. It is also known that epigenetic mechanisms of gene expression are of major importance in the development and maintenance of AUD. The consumption of alcohol induces changes in DNA methylation, histone modifications, and the expression of ncRNAs [70]. The study of alcohol-induced changes in the brain could help in the development of new diagnostic and therapeutic options for AUD [70].

### 6.8. Use of Substances Other Than Alcohol

Substance use disorder (SUD) involves the prolonged use and abuse of substances such as cannabis, opioids, sedative-hypnotics, central nervous system stimulants, and tobacco. SUD is characterized by abnormal and persisting changes in gene expression in the brain. Substances that are abused, and stress and mood disorders act on common neuronal and molecular pathways, especially in the brain reward circuitry system. This system includes dopaminergic neurons in the ventral tegmental area of the midbrain and their postsynaptic targets in the medial prefrontal cortex and nucleus accumbens [71]. It is now known that epigenetic mechanisms such as DNA methylation and histone modifications play important roles in transcriptional and behavioral responses in SUD. Several environmental factors that increase an individual’s vulnerability to SUD can act epigenetically in the brain. A good understanding of the role of epigenetics in SUD could help in the prevention and treatment of SUD by providing diagnostic biomarkers and novel drugs [71].

### 6.9. Microbiota

Microorganisms, or microbes, live in close proximity and association with virtually all animals and plants. The microbiota refers to the collection of microorganisms living in and on mammalian organisms. Microbiota comprise bacteria, viruses, fungi, and archaea, which are single-celled organisms similar to bacteria. The microbiota inhabiting humans are thought to be made of more than 99% in volume and genome of bacteria. The weight of the microbiota in the gastrointestinal tract amounts to many hundred grams [72].

The microbiota epigenetically as well as non-epigenetically influences normal health and disease states, including psychiatric disorders [72]. With regard to epigenetic effects, there is evidence, for example, that the microbiota via short chain fatty acids (SCFA) can inhibit HDACs and exert mild physiological effects. In this context, Li et al. [73] studied 6 case-control 16S amplicon sequencing datasets for psychiatric disorders, including a total of 430 subjects, and compared patterns of microbial composition across these studies. It was found that different psychiatric disorders show similar overall shift patterns. Significant overall patterns of shift were found between SZ and anorexia nervosa, and between SZ and autism. The authors identified six genera within the order Clostridales that were significantly decreased in many psychiatric disorders. The authors found that depletion of the Clostridales was associated with malfunction of amino acid and carbohydrate metabolism, and a reduction in SCFA. The authors inferred from their data that decreased amino acid metabolism and SCFA may have contributed to the development of the psychiatric disorders. 

### 6.10. Prenatal and Postnatal Infections

Prenatal and postnatal infections have been associated with psychiatric disorders [4]. For example, prenatal exposure to influenza epidemics has been reported to increase the risk of developing SZ. Postnatal exposure to AIDS has been associated with AD and mania. Postnatal exposure to Lyme disease, caused by *Borrelia burgdorferi*, has been associated with anxiety. One of the ways by which prenatal and postnatal infections may increase the predisposition to psychiatric disorders is via epigenetic mechanisms. Altered immunological mechanisms involving dysregulated epigenetic mechanisms of gene expression may contribute to the disease process [74].

## 7. Reversal of Genome–Environment Interactions in the Treatment of Psychiatric Disorders

As is clear from the preceding sections, epigenetic mechanisms of gene expression are dysregulated in psychiatric disorders, and thereby could be contributing to the pathogenesis of these disorders. Many, if not all, treatment modalities used in the treatment of psychiatric disorders at least partly, if not entirely, act by altering epigenetic mechanisms of gene expression. Thus, it is possible that these treatments undo what the disease process has done to patients. In the broad sense, the epigenetic effects of these treatments can be considered to be environmental factors interacting with the genome in patients with psychiatric disorders in a reverse manner and thereby alleviating symptoms of the disorders. Given below is how such factors can modify the epigenetic mechanisms of gene expression in the treatment of psychiatric disorders (Table 2).

### 7.1. Drugs

Many of the currently used psychotropic drugs including antipsychotics, anxiolytics, antidepressants, and mood stabilizing drugs are known to alter epigenetic mechanisms that regulate gene expression [75,76]. However, epigenetic actions may not be the main way by which these drugs act in the therapy of these disorders [76]. Regarding epigenetic effects, the antipsychotics clozapine and olanzapine have HDAC inhibiting activity. The antidepressant imipramine inhibits HDAC and histone demethylation.

The antidepressant fluoxetine inhibits HDAC and histone trimethylation. The mood stabilizing drug valproic acid is well known to inhibit HDAC. Indeed, recently it was shown that valproate reverses mania-like behavior in mice by inhibiting HDAC2 in preference to other HDACs [77]. 

In addition to the drugs discussed above, drugs with more specific epigenetic actions are being investigated for the treatment of psychiatric disorders. For instance, HDAC inhibitors are being studied for treating disorders such as MDD [78,79]. The naturally occurring compounds L-methylfolate and S-adenosylmethionine which increase DNA methylation are being studied for treating psychiatric disorders such as MDD [80,81] and SZ [81]. In this context, Roffman et al. [82] in a randomized controlled trial showed that L-methylfolate demonstrated significantly better treatment effects in patients with SZ in comparison to controls. In another study, Nierenberg and co-workers [83] showed that in patients with bipolar depression L-methylfolate combined with usual treatment showed beneficial effects in decreasing depressive symptoms.

### 7.2. Psychotherapy

Psychotherapy is a way to help patients with a broad variety of psychiatric disorders. Psychotherapy can help eliminate or control troubling symptoms so that a patient can function better, and it can increase wellbeing and healing. There is increasing and growing evidence that an important way by which psychotherapy exerts its beneficial actions in the therapy of psychiatric disorders is by modifying brain epigenetic mechanisms of gene expression [36,84,85]. Psychotherapy causes changes in neural circuitry and neurotransmission that correlate with changes in cell and genomic function [86]. There is increasing evidence that psychotherapy promotes an epigenetic fingerprint in the brain that aids the resilience to stress [86]. The data to date have been obtained from the study of epigenetic changes in peripheral blood. For instance, Schiele and colleagues, [87] in a sample of unmedicated female patients with obsessive-compulsive disorder and age- and sex-matched controls, studied DNA methylation of the gene coding for monoamine oxidase-A (MAO-A) from DNA obtained from whole blood and response to cognitive behavior therapy (CBT). Prior to therapy, the MAO-A gene methylation levels in the patients was significantly lower than in controls. After the starting of CBT, clinical improvement was found to significantly correlate with an increase in MAO-A gene methylation levels. More data is, however, needed before such changes can be used as biomarkers of response to psychotherapy [86]. 

### 7.3. Electroconvulsive Therapy

Electroconvulsive therapy (ECT) is a procedure performed under mild general anesthesia in which small electric currents are passed through the brain, intentionally triggering a brief seizure. ECT causes changes in the brain that can quickly reverse symptoms of certain psychiatric disorders. It is used in the management of psychiatric disorders such as MDD, manic episodes, and SZ. The induction of a bilateral generalized seizure is needed for the beneficial effects of ECT. Positron emission tomography (PET) studies have shown that during seizures cerebral blood flow, use of glucose and oxygen, and permeability of the blood-brain barrier increase. ECT also causes changes in virtually every neurotransmitter system. ECT is known to causes changes in epigenetic mechanisms of gene expression [88], and a recent epigenome-wide study of the actions of ECT in 34 depressed patients elucidated many potential CpG sites involved in the response to ECT [89]. More needs to be examined in this area of research.

### 7.4. Physical Exercise

Physical exercise has been shown to be beneficial for not only physical health and wellbeing but also mental health and wellbeing. Patki et al. [90] showed that epigenetic mechanisms such as histone acetylation of H3 and changes of methyl-CpG-binding in the hippocampus may take part in the rescue effects of exercise in social defeat-induced behavioral deficits in rats. In a cross-sectional study in the USA of a sample of 1.2 million individuals aged 18 years or older, it was shown that physical exercise significantly associated with better mental health and wellbeing [91]. It was also shown that specific types, durations, and frequencies of exercise could be better clinical targets than others for diminishing mental health burden [91]. More recently, Grasdalsmoen et al. [92] examined the correlation between physical exercise frequency, intensity, and duration and mental health issues among 50,000 university students in Norway. The authors showed that physical exercise associated negatively with all measures of mental health problems and suicidality in a dose-dependent manner.

One way by which physical exercise may help regarding mental health and wellbeing is by altering epigenetic mechanisms of gene expression in the brain. This is because there is evidence that exercise causes epigenetic regulation of metabolic processes in the body, inflammatory processes, and the aging process [93]. Changes in concentrations of metabolites associated with exercise such as oxygen and the tricarboxylic acid-associated metabolites 2-oxoglutarate, 2-hydroxyglutarate, and ß-hydroxyglutarate can change the function of many enzymes involved in regulating epigenetic mechanisms of gene expression [93]. In the brain, exercise modulates the activity of BDNF and Ca^2+^/calmodulin-dependent protein kinase II (CAMKII). The phosphorylated (activated) form of CAMKII quickens the phosphorylation of cyclic AMP response element-binding protein (CREB). Phosphorylated CREB can recruit CREB-binding protein, which has histone acetyl transferase increasing effects. In the brain exercise causes alterations in DNA methylation, histone modifications, and miRNAs, which can have useful effects on functions of the brain such as cognition and memory [94].

## 8. Conclusions

Several environmental factors act on the genome to influence the pathogenesis of psychiatric disorders. Epigenetic mechanisms of gene expression including DNA methylation, histone modifications, and non-coding RNA-mediated regulation of gene expression are a major, if not the only, link between environmental factors and the genome. For a long time, the way by which environmental factors were involved in the pathogenesis of psychiatric disorders was a mystery. The advent of research on the role of epigenetic mechanisms of gene expression in these disorders is helping to clarify many issues regarding the role of environmental factors in these disorders. Research on the role of epigenetics in these disorders could help in the diagnosis of these disorders by providing biomarkers, and in the treatment of these disorders by facilitating the use of epigenetic drugs.

## Figures and Tables

**Table 1:** Environmental factors interacting epigenetically with the genome in the pathogenesis of psychiatric disorders.

Social determinants of mental health including early life adversity and early life stress
Maternal prenatal stress
Poverty
Migration
Urban dwelling
Pregnancy and birth complications
Alcohol use
Substance use other than alcohol
Microbiota
Prenatal and postnatal infections

**Table 2:** Environmental factors interacting epigenetically with the genome to reverse psychiatric disorders.

Psychotropic drugs
Psychotherapy
Electroconvulsive therapy
Physical exercise

## Data Availability

Not applicable.
